# Effect of farrowing room temperature adjustment on total electrical energy consumption and sow and litter performance

**DOI:** 10.1007/s11250-026-05234-z

**Published:** 2026-07-20

**Authors:** Jacson Eduardo Bittencourt, Carolini Schultz, Vanessa Peripolli, Monike Willemin Quirino, Luciana Foppa, José Luiz Corezzolla, Gustavo Freire Resende Lima, Fabiana Moreira, Thomaz Lucia, Rafael da Rosa Ulguim, Ivan Bianchi

**Affiliations:** 1 MBRF, Concórdia, Santa Catarina 89700-000 Brasil; 2 Instituto Federal Catarinense, Araquari, Santa Catarina 89245-000 Brasil; 3 Iowa State University, 1221 Kildeen Hall, Iowa, USA; 4 Faculdade de Veterinária, Universidade Federal de Pelotas, Pelotas, Rio Grande do Sul 96160-000 Brasil; 5 Faculdade de Veterinária, Universidade Federal do Rio Grande do Sul, Porto Alegre, Rio Grande do Sul 91540-000 Brasil

**Keywords:** Swine housing, Temperature management, Electrical energy consumption, Sow performance

## Abstract

Effective management of farrowing room temperature optimizes production costs by influencing sow feed intake, piglet growth, and long-term productivity, making it a crucial factor for farmers. This study aimed to evaluate the impact of a 2 °C increase in the temperature setpoint in farrowing rooms equipped with a crossflow evaporative cooling system on total electrical energy consumption and sow and litter performance. Sows (parities 1 to 4) were allocated to two temperature treatments. In T23–20 (*n* = 232), room temperature was set to 23 °C during farrowing week and reduced by 1 °C per week, reaching 20 °C at weaning. In T25–22 (*n* = 213), temperatures were managed similarly, ranging from 25 °C to 22 °C. Total electrical energy consumption was 7,724 kWh for T23–20 and 6,151 kWh for T25–22, a 20.4% reduction in the energy consumption with the higher temperature threshold. However, sows in T23–20 had greater average lactation feed intake (6.5 ± 0.1 kg/day vs. 6.3 ± 0.1 kg/day; *P* = 0.03) and litter weight gain adjusted to 26 days (67.8 ± 1.3 kg vs. 62.0 ± 1.4 kg; *P* < 0.01) compared to T25-22. Pre-weaning mortality was not affected by treatment (7.9 ± 0.6% vs. 9.1 ± 0.7%; *P* = 0.23). Parity had a significant effect on sow body weight at farrowing and at weaning, sow lactation feed intake, and piglet average daily gain (*P* < 0.0001). Even small adjustments to the temperature setpoint of farrowing rooms can influence sow feed intake and piglet growth. Increasing the temperature by 2 °C reduced total electrical energy consumption by 20.4% (US$1.00/sow), but the economic losses associated with reduced piglet weaning gain were estimated at US$5.28/litter/sow. Therefore, adopting short- and long-term strategies to maintain cooler farrowing environments and respect the thermal needs of the sow is economically advantageous.

## Introduction

The Brazilian pork industry has achieved substantial productivity gains in recent years, driven by advances in genetics, nutrition, biosecurity, and improvements in housing and management systems. These developments have positioned Brazil as a leading player in the international market (CIAS 2025). Additionally, Brazilian commercial farms have consistently shown improved reproductive performance (Agriness 2026), reflecting the increasing efficiency and technical progress of the sector. However, as production intensifies, there is a growing need to balance productivity with the efficient use of resources. Therefore, implementing sustainable housing strategies that integrate animal welfare, environmental control, and economic viability is essential for maintaining this growth.

Proper management of farrowing rooms is crucial for ensuring the quality of piglets produced and maintaining the body condition of sows for their subsequent reproductive cycles. However, achieving an optimal thermal environment in the farrowing room can be challenging, as it accommodates two different categories of animals that have distinct thermal needs. Newborn piglets are vulnerable to cold stress, with their thermal comfort zone ranging from 32 to 34 °C during their first day of life. They require heating sources to maintain this temperature range (Herpin et al. 2002; Vande Pol et al. 2021), and exposure to temperatures below 25 °C increases the risk of pre-weaning mortality due to hypothermia, starvation, and crushing by the sow (Stansbury et al. 1987; Vande Pol et al. 2021).

Lactating sows have a significantly lower thermoneutral zone compared to their piglets, ranging from 12 to 22 °C (Black et al. 1993). Due to their high metabolic heat production and limited sweat gland function, they are particularly susceptible to heat stress (Rodrigues et al. 2010; Cabezón et al. 2017). Consequently, lactating sows require cooling systems for optimal performance. This issue is particularly important in Brazil, where most swine production systems are situated in subtropical regions that require temperature control, especially during the summer months when ambient temperatures can rise significantly. When lactating sows are exposed to temperatures above 25 °C, their thermoregulatory mechanisms can become overwhelmed, leading to reduced feed intake and decreased milk yield (Quiniou and Noblet 1999; Ribeiro et al. 2018). These physiological responses to heat stress are associated with slower litter growth and may also influence sows’ subsequent reproductive performance and overall longevity.

Compared to farrowing rooms that utilize natural ventilation, facilities equipped with automated cooling systems contribute to improved sow welfare (Justino et al. 2014), resulting in heavier piglets at weaning (Ribeiro et al. 2018; Ricci et al. 2018; Mendes et al. 2020) and lead to lower pre-weaning mortality rates (Leite et al. 2025). Consequently, the adoption of automated climate control technologies has increased in Brazil. Nonetheless, there remains a lack of practical guidance on adjusting cooling systems to maximize productivity while minimizing costs with electrical energy consumption under commercial conditions. The hypothesis tested was that increasing farrowing room temperature could reduce total electrical energy costs without adversely affecting animal performance. Therefore, this study aimed to evaluate the effect of a 2 °C adjustment in the activation temperature of the farrowing room cooling system on sow and litter performance, as well as total electrical energy consumption, in a commercial swine farm.

## Materials and methods

### Study location and facilities

The study was conducted at a commercial farm in southern Brazil (27°13’24.68” S, 52°23’34.09” W, elevation 612 m) between November and January, corresponding to late spring and early summer in the Southern Hemisphere. According to the Köppen climate classification, the region has a temperate humid climate (Cfb), characterized by mild summers and well-distributed precipitation throughout the year, with a mean annual temperature of 19 °C and an average annual precipitation of 1,800 mm.

**Fig. 1:**
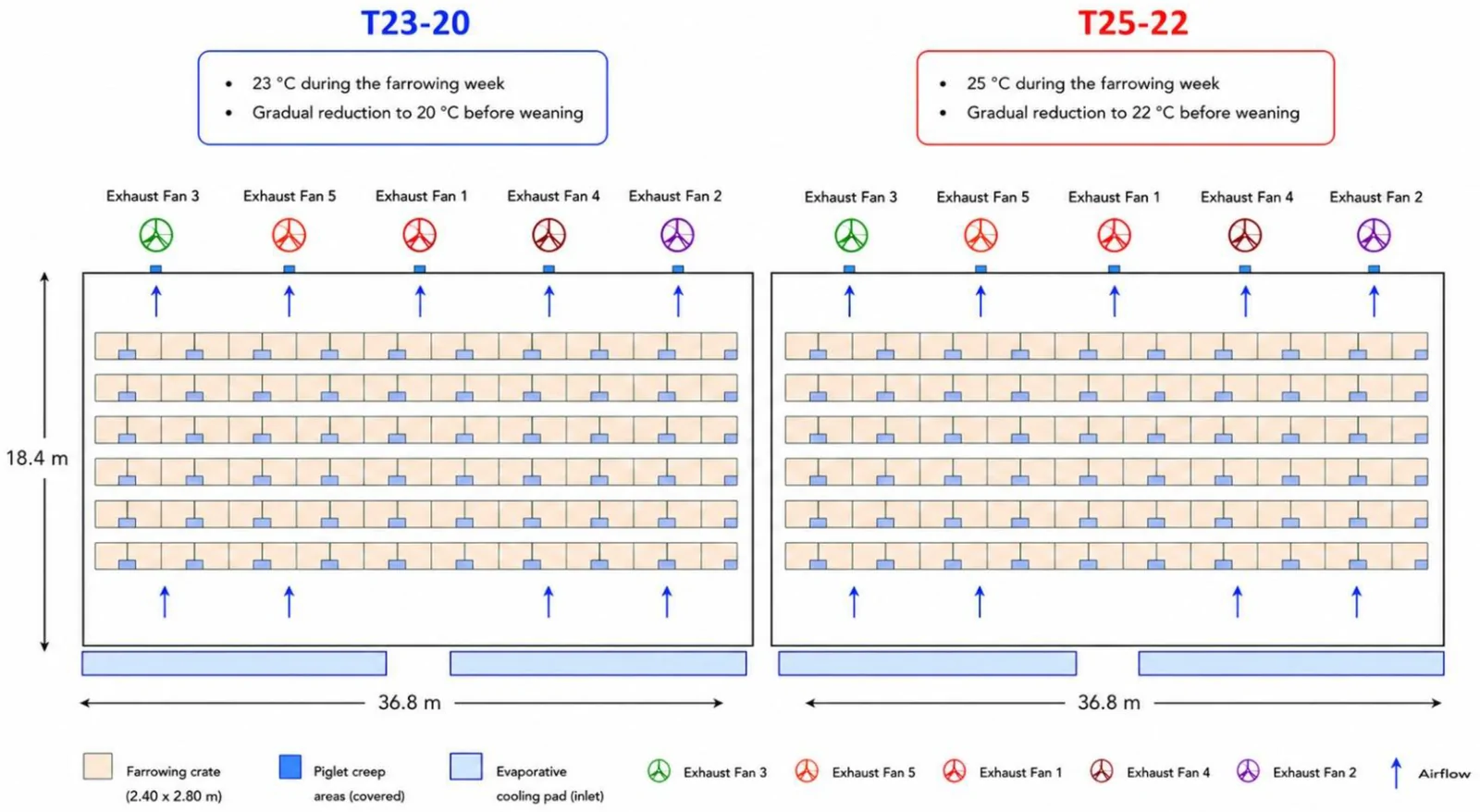
Floor plan of the farrowing rooms, containing 108 crates (2.40 × 2.80 m) arranged in six rows. Each crate included a covered area dedicated to the piglets. Room 1: T23-20: The setpoint of Exhaust Fan I was adjusted to 23 °C during the farrowing week, with a gradual reduction to 20 °C in the days preceding weaning. Room 2: T25-22: The setpoint of Exhaust Fan I was adjusted to 25 °C during the farrowing week, with a gradual reduction to 22 °C in the days preceding weaning

The experiment included two farrowing rooms, each measuring 19.7 m in width and 34.6 m in length, equipped with a ceiling-mounted deflector positioned at a height of 2.80 m. Each room contained 108 farrowing crates (2.40 × 2.80 m) arranged in six rows, each with a covered area dedicated to the piglets (Fig. 1). Piglets had a covered area with a concrete floor heated by an electric resistance system, with the floor temperature maintained at 30–32 °C during the first week of housing and gradually reduced to 25–26 °C by the end of a 26-day sucking period.

The rooms were equipped with crossflow evaporative cooling systems (Smaai 04, Inobran, Brazil). The digital controller operated six sets of exhaust fans (0.84 kW / 1.12 Hp, 50” diameter with an extraction capacity of 33,000 m³/h each), allowing air velocities between 0 and 1.0 m/s across six distinct stages. The activation temperature of the cooling system differed by 2 °C between treatments (Table 1). The cooling system operated sequentially: when the room temperature exceeded the initial temperature setpoint, Exhaust Fan 1 was activated, followed by Exhaust Fan 2, the evaporative pad, and then Exhaust Fans 3, 4, and 5 (Fig. 1). Farrowing room temperature was dynamically regulated, with higher values maintained during the farrowing week and gradually reduced by 1 °C per week after. Relative humidity ranged from 40 to 90% during the study period.

In the T23–20 treatment room (*n* = 232), the temperature setpoint for Exhaust Fan I was initially 23 °C during the farrowing week and was decreased by 1 °C per week, reaching a final temperature of 20 °C before weaning. In the T25–22 treatment room (*n* = 213), the initial setpoint was 25 °C during the farrowing week and was gradually reduced to 22 °C before weaning.

Importantly, the treatments differed exclusively in the setpoint of Exhaust Fan I. All subsequent ventilation stages (Exhaust Fans II-V) and the evaporative cooling system were operated using the same temperature thresholds in both rooms, ensuring that the primary experimental contrast was restricted to the initial ventilation stage.

Room temperature was manually monitored and recorded three times daily (8:00, 14:00 and 18:00). Due to data recording issues, temperature data from the first batch of the T25–22 treatment were unavailable. As this resulted in an unbalanced dataset across treatments and batches, inferential statistical analyses could be biased or unreliable. Therefore, temperature values were summarized using descriptive statistics only.

Total electrical energy consumption (kWh) was continuously recorded for each farrowing room throughout the experimental period using cumulative meter readings. Daily energy consumption (kWh) was calculated as the difference between consecutive readings obtained at 10:00 on each housing day. Data were screened to exclude implausible values, such as negative values and extreme readings associated with meter resets, and only valid daily observations were retained for analysis.

**Table 1:** Temperature programs for the farrowing rooms equipped with cross-flow evaporative cooling systems

Housing period (day)	Exhaust Fan I	Exhaust Fan I	Exhaust Fan II	Evaporative Pad	Exhaust Fan III	Exhaust Fan IV	Exhaust Fan V
	T23-20	T25-22	T23-20 and T25-22				
Housing − 1st farrowing	21	23	24.5	26.2	26.5	28	29.5
1–7	23	25	26.5	28.2	28.5	30	31.5
8–15	22	24	25.5	27.2	27.5	29	30.5
16–22	21	23	24.5	26.2	26.5	28	29.5
23 -Weaning	20	22	23.5	25.2	25.5	27	28.5
Air velocity (m/s)	0.25	0.25	0.45	0.45	0.60	0.75	1.00

T23-20: The setpoint of Exhaust Fan I was adjusted to 23 °C during the farrowing week, with a gradual reduction to 20 °C in the days preceding weaning

T25-22: The setpoint of Exhaust Fan I was adjusted to 25 °C during the farrowing week, with a gradual reduction to 22 °C in the days preceding weaning

### Experimental design

A total of 445 crossbred sows (Landrace × Large White) and their litters were evaluated over three consecutive batches on a farm located in southern Brazil (27°13’24.68” S, 52°23’34.09” W, altitude 612 m), a region with a humid mesothermal climate. Each batch included sows from both treatment groups, which were housed simultaneously in two paired farrowing rooms with similar structural characteristics and management conditions. Each room was assigned to a single treatment throughout the study; therefore, the room was considered the experimental unit.

Within each batch, sows were allocated to treatments in a balanced manner according to parity (1 to 4) to minimize potential confounding effects and ensure comparable groups. Animals were moved to their designated farrowing rooms seven days before the expected farrowing date.

Measurements were collected repeatedly over time during the lactation period. During the prepartum period, sows received 2.8 kg of feed per day; after farrowing, feed was provided *ad libitum*. Daily feed intake was determined by manually recording the amount of feed offered and orts for each sow. Water was available *ad libitum* throughout the experiment.

Sow body weight was recorded upon entry and exit of the farrowing room. Postpartum sow weight was estimated using Eq. 1. from Mallmann et al. (2018): $\:\gamma\:=13.03+(0.93\times\:{\beta\:}_{1})-(1.23\times\:{\beta\:}_{2})$, where $\:{\beta\:}_{1}$is the body weight before farrowing and $\:{\beta\:}_{2}$is the total number of piglets born (live plus stillborn). Sow body condition score (BCS) was assessed at farrowing and at weaning using the caliper scoring system (scale 1–29; Knauer and Baitinger 2015). Backfat thickness (mm) was recorded using a Renco Lean-Meater (MS Schippers, Netherlands).

All farrowings were monitored, and the total born alive, stillborn, and mummies were recorded. Piglets remained with their dams for the first 24 h to ensure adequate colostrum intake. Thereafter, cross-fostering was performed to equalize litter according to the number of viable teats per sow. Throughout lactation, piglets had *ad libitum* access to water; however, no creep feeding was provided. Sow voluntary feed intake (kg), litter weight gain (kg), and piglet average daily gain (ADG, g/d) were adjusted to a 26-d lactation period.

### Statistical analysis

Analyses were conducted using SAS (version 9.3; SAS Institute Inc., Cary, NC, USA) and R (version 4.3.2; R Foundation for Statistical Computing, Vienna, Austria). The farrowing room was the experimental unit for the temperature treatment.

Continuous outcomes were analyzed using mixed-effects models (PROC MIXED). For animal performance variables, the model included treatment, parity, and their interaction as fixed effects, with batch included as a random effect. For energy consumption, the model included treatment, housing day, and their interaction as fixed effects, with batch included as a random effect. Because measurements were collected repeatedly over time, appropriate covariance structure was specified to account for within-room correlations. Least-squares means were estimated, and pairwise comparisons were adjusted using Tukey’s test.

Categorical outcomes were analyzed using generalized linear mixed models (PROC GLIMMIX), specifying the appropriate distribution and link function, with batch included as a random effect. Associations between variables were evaluated using Pearson’s correlation coefficient.

Model assumptions were assessed by inspection of residual plots and by testing normality (Shapiro–Wilk) and homogeneity of variance (Levene’s test). All assumptions were met. Data processing and figure generation were performed in R using the tidyverse package. Statistical significance was set at *p* < 0.05.

## Results

### Energy consumption

**Fig. 2:**
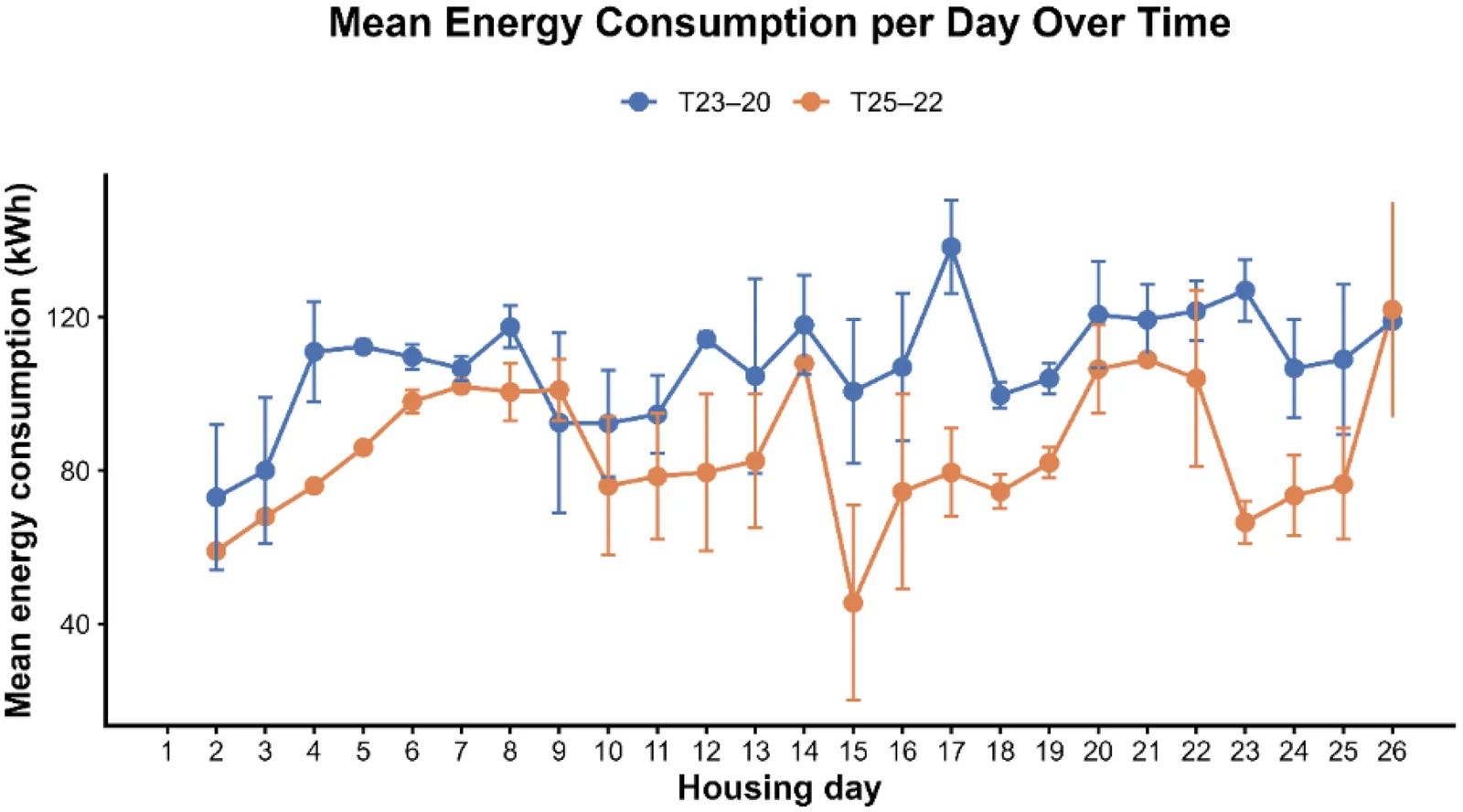
Mean daily electrical energy consumption (kWh) in farrowing rooms under two temperature programs (T23-20 and T25-22) over the 26-day experimental period. Values represent means ± standard error

Treatment had a strong effect on energy consumption (*P* < 0.001). There was no evidence of a treatment per day interaction (*P* = 0.80), indicating that the treatment effect remained consistent over time. The day did not affect energy consumption (*P* = 0.06), although a trend toward variation across days was observed. Estimated daily electrical energy consumption was higher in T23-20 than in T25-22 (108 ± 3.57 vs. 86 ± 4.63 kWh, respectively), corresponding to a difference of 22 ± 4.74 kWh per day (~ 20.4% points; *P* < 0.0001; Fig. 2).

### Temperature variables

**Fig. 3:**
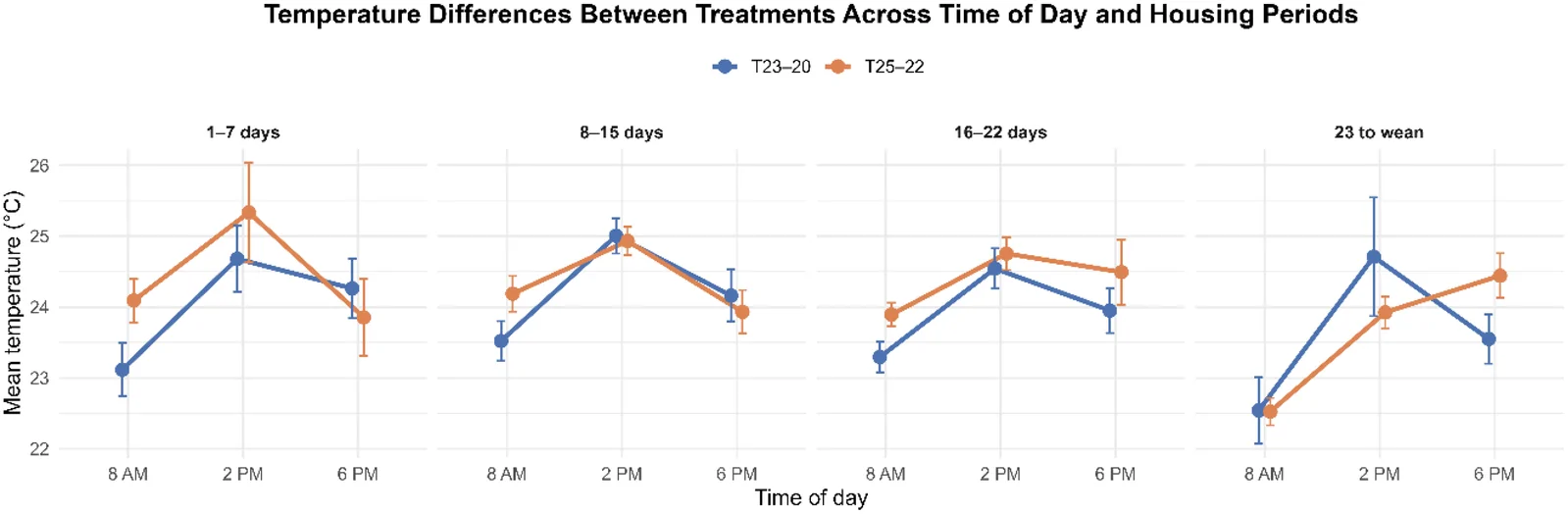
Mean room temperature varied according to time of day (8 AM, 2 PM, and 6 PM), housing period (1–7 days, 8–15 days, 16–22 days, and 23 to wean), and temperature programs (T23-20 and T25-22). Values represent means ± standard error

Temperature varied across treatments, housing periods, and time of day (Fig. 3). A clear diurnal pattern was observed in both treatments, with the lowest values recorded at 8:00 AM and the highest at 2:00 PM. Mean temperatures across housing periods were: 23.20 ± 0.16 °C (T23-20) and 23.82 ± 0.14 °C (T25-22) at 8:00 AM; 24.75 ± 0.21 °C (T23-20) and 24.82 ± 0.20 °C (T25-22) at 2:00 PM. An exception to this pattern was observed in the T25-22 treatment during the last housing period, when the mean temperature at 6:00 PM exceeded that recorded at 2:00 PM.

Differences between treatments were more pronounced during the morning, with the highest mean difference between T23-20 and T25-22 observed at 8 AM from 1 to 7 day housing period (+ 0.96 ± 0.49 °C) when overall temperatures were lower and environmental control relied primarily on Exhaust Fan I, which was the only component with distinct setpoints between treatments. As the day progressed, temperatures increased, and environmental control relied more on higher ventilation stages, such as Exhaust Fan II and Exhaust Fan III.

### Animal performance

No treatment per parity interaction was observed on sow and litter performance (*P* > 0.05). Temperature did not affect sow body weight at weaning (*P* = 0.45), pre-weaning mortality (*P* = 0.23), or weaned litter size (*P* = 0.10), but it influenced piglet weight gain (Table 2). Compared with sows kept in T25-22, those in T23-20 weaned heavier piglets and had higher piglet ADG (*P* = 0.0001). Litter weight gain adjusted to 26 lactation days was 67.8 ± 1.3 kg for T23-20 and 62.0 ± 1.4 kg for T25-22 (*P* < 0.01).

Because feed allowance during the prepartum period was restricted, no difference in prepartum feed intake was observed between treatments (*P* = 0.20; Table 3). Feed was offered *ad libitum* after farrowing, and sows in T23-20 had higher daily average lactation feed intake (*P* = 0.03) and higher postpartum feed intake adjusted to 26 lactation days than those in T25-22 (*P* = 0.03).

The main effects of parity on sow reproductive performance, sow feed intake, and litter outcomes are shown in Table 4 (*P* < 0.05). Parity-4 sows farrowed larger litters and had higher total (*P* = 0.01) and born alive litter size (*P* = 0.02) than parity-2 sows. Pre-weaning mortality was higher for parity-3 sows than parity-2 and parity-4 sows (*P* = 0.05). Interestingly, primiparous sows weaned larger litters than parity-4 sows (*P* = 0.01), and fewer piglets were assigned to parity-4 sows after cross-fostering, due to their lower number of functional teats. Considering litter size after cross-fostering, sows from parities 1–4 weaned 90.8%, 92.8%, 89.2%, and 92.3% of their piglets, respectively.

Adjusted body weight at both farrowing and weaning increased progressively from parities 1 to 4. Primiparous sows had the lowest average daily feed intake, whereas parity-4 sows had the highest intake (Table 4). Litter weight gain was higher for parity-4 sows than for primiparous sows (*P* < 0.01). There was no difference in piglet lactation ADG between sows of parities 1 and 2 (*P* > 0.05), with the highest ADG observed in piglets from parity-4 sows.

Positive correlations (*P* < 0.001) were observed among most traits (Table 5). Adjusted postpartum weight showed a strong correlation with weaning weight (*r* = 0.862). Moderate positive correlations were observed between BCS at weaning and weaning weight (*r* = 0.481), as well as between BCS at both farrowing and weaning (*r* = 0.433). Backfat thickness at farrowing was moderately correlated with BCS at farrowing (*r* = 0.519) and backfat thickness at weaning (*r* = 0.516), indicating that sows tend to maintain BCS and body reserves throughout lactation.

**Table 2:** Sow and litter performance under the farrowing room temperature program treatments (means ± SEM)

Parameter	Temperature Program		*P*-value		
	T23-20	T25-22	Treatment	Parity	Treatment*Parity
*Females (n)*	232	213	-	-	-
Parity	2.8 ± 0.1	2.9 ± 0.1	-	-	-
Lactation length (d)	26.0 ± 0.1	26.0 ± 0.1	-	-	-
*Litter size*					
Total born (n)	14.3 ± 0.2	14.1 ± 0.3	0.45	0.01	0.14
Born alive (n)	13.3 ± 0.2	13.2 ± 0.2	0.70	0.02	0.30
Stillborn (%)	5.38 ± 0.5	4.57 ± 0.5	0.25	0.19	0.61
Mummies (%)	0.9 ± 0.2	1.0 ± 0.2	0.84	0.26	0.69
Cross-fostered (n)	12.3 ± 0.1	12.2 ± 0.1	0.30	< 0.0001	0.83
Pre-weaning mortality (%)^*^	7.9 ± 0.6	9.1 ± 0.7	0.23	0.04	0.31
Weaned piglets (n)	11.3 ± 0.1	11.0 ± 0.1	0.10	0.01	0.44
*Sow body weight*					
At farrowing (kg)^**^	214.9 ± 1.5	216.0 ± 1.7	0.74	< 0.0001	0.14
At weaning (kg)	222.6 ± 1.9	222.8 ± 2.2	0.45	< 0.0001	0.22
*Litter weight*					
Cross-fostered 24 h post-farrowing (kg)	15.2 ± 0.1	15.0 ± 0.1	0.30	< 0.0001	0.83
Weight gain during lactation (kg)^***^	68.8 ± 1.3	62.0 ± 1.4	< 0.01	< 0.0001	0.97
Piglet weight at weaning (kg)^***^	7.2 ± 0.1	6.8 ± 0.1	0.01	< 0.0001	0.94
ADG (g/day)^***^	228.0 ± 3.7	213.1 ± 3.9	0.01	< 0.0001	0.95

^*^With respect to the number of piglets that were cross-fostered

^**^Adjusted according to Malmann et al. (2018)

***Adjusted to 26 lactation d (kg)

T23-20: The setpoint of Exhaust Fan I was adjusted to 23 °C during the farrowing week, with a gradual reduction to 20 °C in the days preceding weaning

T25-22: The setpoint of Exhaust Fan I was adjusted to 25 °C during the farrowing week, with a gradual reduction to 22 °C in the days precedingweaning

Fan I was adjusted to 25 °C during the farrowing week, with a gradual reduction to 22 °C in the days preceding weaning.

**Table 3:** Feed intake of sows under the farrowing room temperature program treatments (means ± SEM)

Parameter	Temperature program		*P*-value		
	T23-20	T25-22	Treatment	Parity	Treatment*Parity
Females (n)	232	213	-	-	-
Total prepartum feed intake (kg)	19.7 ± 0.6	19.0 ± 0.6	0.20	0.23	0.71
Average lactation feed intake (kg/day)	6.5 ± 0.1	6.3 ± 0.1	0.03	< 0.0001	0.85
Postpartum feed intake adjusted to 26 lactation d (kg)	169.6 ± 1.6	164.5 ± 1.7	0.03	< 0.0001	0.84

T23-20: The setpoint of Exhaust Fan I was adjusted to 23 °C during the farrowing week, with a gradual reduction to 20 °C in the days preceding weaning

T25-22: The setpoint of Exhaust Fan I was adjusted to 25 °C during the farrowing week, with a gradual reduction to 22 °C in the days preceding weaning

**Table 4:** Reproductive performance, feed intake, and litter performance of sows according to parity (means ± SEM)

Parameter	Parity				
	1 (*n* = 80)	2 (*n* = 82)	3 (*n* = 98)	4 (*n* = 185)	*P*-value
*Litter size*					
Total piglets born (n)	13.5 ± 0.4ᵃᵇ	13.3 ± 0.4ᵇ	14.3 ± 0.4ᵃᵇ	14.7 ± 0.3ᵃ	0.01
Piglets born alive (n)	12.7 ± 0.4ᵃᵇ	12.5 ± 0.4ᵇ	13.5 ± 0.3ᵃᵇ	13.6 ± 0.2ᵃ	0.02
Stillborns (%)	5,1 ± 0.8	5.1 ± 0.8	3.8 ± 0.7	5.7 ± 0.5	0.19
Mummies (%)	0.9 ± 0.3	0.6 ± 0.3	1.0 ± 0.3	1.2 ± 0.2	0.23
Cross-fostered (n)	12.8 ± 0.2ᵃ	12.3 ± 0.2ᵇ	12.6 ± 0.1ᵃᵇ	11.8 ± 0.1ᵇ	< 0.0001
Pre-weaning mortality (%)^*^	9.0 ± 1.4ᵃᵇ	7.3 ± 1.1ᵃ	10.8 ± 1.0ᵇ	7.6 ± 0.7ᵃ	0.05
Weaned piglets (n)	11.6 ± 0.2ᵃ	11.4 ± 0.2ᵃᵇ	11.3 ± 0.2ᵃᵇ	10.9 ± 0.1ᵇ	0.01
*Sow body weight*					
Body weight at farrowing (kg)^**^	184.2 ± 1.9ᵃ	204.0 ± 1.9ᵇ	220.5 ± 1.7ᶜ	231.2 ± 1.2ᵈ	< 0.0001
Body weight at weaning (kg)	181.1 ± 2.2ᵃ	208.8 ± 2.2ᵇ	228.6 ± 2.0ᶜ	243.6 ± 1.5ᵈ	< 0.0001
Daily lactation feed intake (kg/day)	5.7 ± 0.1ᵃ	6.5 ± 0.1ᵇ	6.6 ± 0.1ᵇ	6.9 ± 0.1ᶜ	< 0.0001
Postpartum feed intake (kg)^***^	149.2 ± 2.6ᵃ	168.4 ± 2.5ᵇ	171.2 ± 2.3ᵇ	179.6 ± 1.7ᶜ	< 0.0001
*Litter weight*					
Litter weight gain (kg)^***^	60.5 ± 2.1ᵃ	65.3 ± 2.1ᵃᵇ	65.5 ± 1.9ᵃᵇ	69.0 ± 1.4ᵇ	< 0.01
Piglet ADG (g/day)^***^	198.5 ± 5.9ᵃ	218.3 ± 5.9ᵇ	221.7 ± 5.4ᵇ	244.3 ± 3.9ᶜ	< 0.0001

^*^With respect to the number of piglets that were cross-fostered

^**^Weight adjustment according to Malmann et al. (2018)

^***^Adjusted to 26 lactation d (kg)

^a,b,c^ Means ± SEM with distinct superscripts indicate significant differences

**Table 5:** Pearson correlation coefficient (r) among parameters of growth performance in lactating sows (*n* = 440)^*^

Parameter	Postpartum adjusted weight (kg)	Weaning weight (kg)	BCS at farrowing (1–29°)	BCS at weaning (1–29°)	Backfat at farrowing (mm)
Weaning weight (kg)	0.862	-	-	-	-
BCS at farrowing (1–29°)	0.306	0.197	-	-	-
BCS at weaning (1–29°)	0.387	0.481	0.433	-	-
Backfat at farrowing (mm)	0.235	0.197	0.519	0.394	-
Backfat at weaning (mm)	0.218	0.233	0.409	0.464	0.516

^*^All correlations were significant (*P* < 0.001)

## Discussion

The present study tested the hypothesis that increasing farrowing room temperature could reduce total electrical energy costs without adversely affecting animal performance. In T25-22, this temperature adjustment resulted in a 20.4% reduction in electrical energy consumption, corresponding to savings of US$204.49 (~ US$1.00/sow) during the evaluated period (based on an electricity price of US$0.13 per kWh). However, these savings were accompanied by reductions in sow voluntary feed intake, piglet ADG during lactation, and weaning weight (–0.4 kg per piglet) compared with T23-20.

To illustrate these effects in economic terms, the observed performance losses could correspond to an estimated gross loss of US$1,301.28 (approximately US$5.28/litter/sow), considering a total of 2,711 piglets housed in T25-22 and a market price of US$1.20 per kg of live weight. Therefore, although energy savings were observed, they were substantially outweighed by the economic impact associated with reduced piglet performance. This trade-off highlights that, under the evaluated conditions, small increases in farrowing room temperature may not be economically advantageous when animal performance losses are considered. In regions with higher electricity prices or during periods when pork prices are lower, the balance between energy savings and production losses may be less pronounced; however, the biological constraints associated with thermal stress would still need to be considered.

The reduction in litter performance was associated with reduced feed intake of sows in the T25-22. Adult pigs have a limited ability to dissipate heat due to their low surface area-to-body volume ratio and poorly functional sweat glands, making them highly susceptible to heat stress (Rodrigues et al. 2010). This vulnerability is even more pronounced in lactating sows, as the high feed intake required for milk production generates additional metabolic heat compared to other animal categories (Brown-Brandl et al. 2014; Cabezón et al. 2017). To maintain euthermia, heat-stressed animals may pant or manipulate drinkers to wet themselves, increasing heat dissipation, and may also reduce metabolic heat production by decreased feed intake (Quiniou and Noblet 1999; Ribeiro et al. 2018; Ricci et al. 2018; Cecil et al. 2025). Although heat stress indicators were not directly measured in the present study, the observed reduction in feed intake under higher environmental temperatures is consistent with a thermoregulatory response.

Because most nutrients consumed by lactating sows are allocated to milk production, reduced feed intake directly impairs litter growth (Strathe et al. 2017). It is estimated that every 1 °C above the upper critical limit of the thermoneutral zone is related to a 0.189 kg/day decrease in sow feed intake, leading to a daily reduction of 0.124 kg/day in milk yield (Dourmad et al. 2022). In the present study, sows in T25-22 exhibited a daily decrease of 0.2 kg/day in feed intake, corresponding to a 3% reduction compared with those in T23-20, which resulted in a 5.8 kg reduction in litter weaning weight in T25-22. This loss is not only an immediate financial concern but also compromises subsequent reproductive performance, as low feed intake during lactation has been linked to decreased farrowing rates and reduced weaned litter size in subsequent litters (Estrada et al. 2024; Rodríguez et al. 2023).

Sow feed intake and piglet ADG during lactation were lower in primiparous than in multiparous sows. Primiparous sows were the only category to lose weight during lactation (~ 3 kg on average) in the present study. However, no interaction was observed between parity and the temperature program, indicating that the increase in temperature that activated the cooling system affected sows of different parities similarly. Under more extreme thermal conditions, by contrast, primiparous sows were more vulnerable to heat stress, exhibiting higher rectal temperatures than multiparous sows (Gourdine et al. 2007). Along with the correlation analysis, these results highlight that although sows can maintain body condition and energy reserves throughout lactation, primiparous sows are a particularly vulnerable subgroup and may require tailored nutritional support, such as higher-energy diets or feed additives, to maintain adequate feed intake and sustain milk production.

Moreover, metabolic heat production in modern-genetics sows is 24–36% higher than in sows from previous decades, highlighting the need to update operational guidelines for temperature control in farrowing rooms (Brown-Brandl et al. 2014; Cabezón et al. 2017). Considering physiological, welfare, and production indicators, it is recommended to maintain farrowing room temperatures between 17 and 21 °C (Cecil et al. 2025). However, in practice, farrowing rooms are often maintained at temperatures above the thermal comfort limits of sows to prioritize piglet comfort, particularly during the first week of life, and microclimate control technologies are either underused or frequently used with improper settings (Cecil et al. 2025; Johnson and Stewart 2025). This indicates that current recommendations and temperature management strategies for farrowing rooms need to be reconsidered.

The present study showed that even slight changes, such as raising the temperature setpoint by 2 °C to activate the cooling system, can lead to immediate negative impacts on piglet growth performance. However, this interpretation should be considered in light of the study’s limitations. Treatments were applied at the farrowing-room level, with each room assigned to a specific temperature program, which did not allow complete separation of treatment effects from potential room-specific factors, such as airflow distribution and equipment performance. Nevertheless, the study was conducted under commercial farm conditions, including a large number of sows (*n* = 445), and repeated over time, providing practical insight into real-world system responses. These findings may support decision-making in commercial systems, although caution is necessary when extrapolating to other environments and management conditions.

In addition to the environmental temperature, relative humidity plays a critical role in determining the effective thermal environment experienced by sows. At higher humidity levels, even slight temperature increases can exacerbate the effects of thermal stress. In the present study, humidity ranged from 40 to 90%, which may have influenced how sows perceived the environmental temperature. High relative humidity impairs evaporative heat loss (Baker 2004), and under such conditions, animals may experience greater thermal stress than indicated by the environmental temperature alone, which, in turn, affects overall performance (Dourmad et al. 2022; Brown-Brandl et al. 2014). This may have contributed to the observed reductions in sow feed intake and piglet growth, even with relatively small differences in temperature setpoints.

Strategies to mitigate the effects of reduced sow feed intake during thermal stress can be implemented at both short- and long-term levels. Short-term strategies include nutritional and management interventions, such as encouraging water consumption, providing diets with higher energy density (Black et al. 1993; Dourmad et al. 2022), and offering additional feed during cooler periods, such as at night (Choi et al. 2019). Long-term strategies involve structural solutions, including roofing and building materials with enhanced thermal insulation and the installation of less common cooling systems, such as drip or snout cooling (Stansbury et al. 1987; Barbari et al. 2007). Another long-term strategy is the selection of genetic lines better adapted to dissipate heat (Byrd et al. 2025).

## Conclusion

Small increases in the temperature setpoint for activating farrowing room cooling systems can reduce sow feed intake and piglet growth. Although a 2 °C increase decreased electrical energy consumption by 20.4%, the associated economic losses due to reduced piglet weaning weight were substantially greater. However, these results should be interpreted in the context of the specific seasonal and regional conditions under which the study was conducted, including climate, electricity costs, and pork prices. Therefore, caution is warranted when extrapolating these findings. Nevertheless, the biological constraints imposed by thermal stress are relevant across production systems and should be considered when designing environmental control strategies in farrowing facilities.

## Data Availability

The data analyzed during the current study are available from the corresponding author on request.

## Declarations

### Generative IA statement

Portions of this manuscript were edited using ChatGPT (GPT-5) and Grammarly solely to improve language clarity and readability. The text was revised by all authors before submission, and all intellectual content, analyses, and conclusions are our own.
